# Associations of depressive symptoms, social engagement and support, and lifestyle behaviors among non-Hispanic black and Hispanic men with chronic conditions in the United States

**DOI:** 10.3389/fpubh.2025.1600818

**Published:** 2025-06-16

**Authors:** Jeong-Hui Park, Caroline D. Bergeron, Michael Ness, Ledric D. Sherman, Ashley L. Merianos, Moka Yoo-Jeong, Cynthia L. Cisneros Franco, Aditi Tomar, Ali Boolani, Chung Lin Kew, Oluyomi Oloruntoba, Matthew Lee Smith

**Affiliations:** ^1^Department of Health Behavior, School of Public Health, Texas A&M University, College Station, TX, United States; ^2^Center for Health Equity and Evaluation Research, Texas A&M University, College Station, TX, United States; ^3^LIFE Research Institute, University of Ottawa, Ottawa, ON, Canada; ^4^School of Human Services, University of Cincinnati, Cincinnati, OH, United States; ^5^School of Nursing, Bouvé College of Health Sciences, Northeastern University, Boston, MA, United States; ^6^Department of Sociology, College of Arts and Sciences, Texas A&M University, College Station, TX, United States; ^7^Department of Health Behavior, Gillings School of Global Public Health, University of North Carolina, Chapel Hill, NC, United States; ^8^Human Performance and Nutrition Research Institute, Oklahoma State University, Stillwater, OK, United States; ^9^Department of Physiology and Pharmacology, Center for Health Sciences, Oklahoma State University, Tulsa, OK, United States; ^10^Center for Community Health and Aging, Texas A&M University, College Station, TX, United States

**Keywords:** depression, depressive symptoms, social engagement, chronic conditions, social support, Hispanic men, Black men

## Abstract

**Introduction:**

Self-management of depressive symptoms is influenced by co-morbidity, social support, and health-related behaviors. Men are less likely to discuss depressive moods and seek healthcare. This study examines factors associated with depressive symptoms among non-Hispanic Black and Hispanic men ages ≥40 years with ≥1 chronic condition in the U. S.

**Methods:**

Data from 1,907 non-Hispanic Black (*n* = 1,117) and Hispanic (*n* = 790) males with chronic conditions were analyzed using logistic regression to assess depressive symptoms, identified as a Patient Health Questionnaire-2 score ≥3. One model was fitted for all men, then separate models were fitted for non-Hispanic Black and Hispanic men, respectively. The models adjusted for sociodemographic, disease characteristics, health status, social engagement and support, and lifestyle behaviors.

**Results:**

In the full model, Hispanic men (OR = 1.39, *p* = 0.017) and those taking more medications (OR = 1.10, *p* = 0.010) were more likely to have depressive symptoms. Social disconnection (OR = 1.65, *p* < 0.001), reliance on others for health management (OR = 1.04, *p* < 0.001), limited activity due to health (OR = 3.15, *p* < 0.001), self-care barriers (OR = 1.16, *p* < 0.001), healthcare frustration (OR = 1.13, *p* < 0.001), prolonged sitting (OR = 1.01, *p* = 0.030), and tobacco use (OR = 1.56, *p* = 0.002) increased likelihood of depressive symptoms. Common and unique factors associated with depressive symptoms were identified in models for non-Hispanic Black and Hispanic men, respectively.

**Conclusion:**

Findings highlight the dynamic interplay between depressive symptoms, social engagement, and lifestyle behaviors among non-Hispanic Black and Hispanic men with complex disease profiles. Efforts are needed to address depressive symptomatology through self-managing conditions, strengthening supportive networks, and alleviating burdens associated with healthcare interactions.

## 1 Introduction

Depression is a widespread chronic condition affecting approximately 10 million Americans annually (1, 2). Non-Hispanic Black and Hispanic individuals tend to experience more severe, prolonged, and debilitating episodes of depression compared to non-Hispanic White individuals (3–8). Several factors may explain this disparity, such as socioeconomic status (e.g., higher rates of poverty, lower rates of health insurance coverage) (9), discrimination (10, 11), and inequitable access and use of mental health care services (9, 12). Non-Hispanic Black and Hispanic individuals were also disproportionately affected by depression during the COVID-19 pandemic (13, 14).

The prevalence and presentation of depressive symptoms differ by sex (14–16), age (17–19), and self-reported physical and mental health status (20–23). Women tend to report more classic depressive symptoms, such as depressed mood, more frequently and intensely than men (24). In contrast, men with depression are more likely to exhibit behaviors such as risk-taking, poor impulse control, and substance misuse (25). Although women have higher overall rates of depression (7, 26), men are more often underdiagnosed (27–30) and undertreated (27, 30). This underdiagnosis and lack of treatment among men can lead to a higher recurrence of depression (31) and an increased risk of suicide attempts and completion (31–34).

Men with chronic conditions are at a heightened risk for depression, particularly when they have multiple chronic conditions (35–39). Lifestyle choices such as physical activity or alcohol consumption can either exacerbate or mitigate the risks associated with both depression and chronic conditions (40–43). Other factors, such as social engagement and support (44, 45), may also play a key role in men’s self-management of depressive symptomatology. Social support is a well-known protective factor to mental health conditions and studies consistently demonstrate a robust link between social support and depressive symptoms in chronically ill individuals, either directly or indirectly through self-management behaviors (46, 47).

Despite the existing literature, less is known about depressive symptoms among non-Hispanic Black and Hispanic men with chronic conditions, subgroups who may face significant disparities in mental health. Given the multifaceted nature of depressive symptomatology management, this study aims to investigate the prevalence of self-reported depression and explore associated factors within a national sample of non-Hispanic Black and Hispanic men aged 40 years and older with one or more chronic conditions in the United States.

## 2 Materials and methods

### 2.1 Participants and procedures

This study utilized a web-based panel survey, a method commonly employed by researchers to reach populations that are hard to access due to high costs and low response rates (48). Specifically, the parent study was conducted through an online survey managed by the Qualtrics panel, with participants completing the questionnaire via the internet. The Qualtrics panel, an opt-in survey platform, is well-suited for studies targeting hard-to-reach populations, providing access to a pre-identified pool of participants who meet specific research criteria. The parent study aimed to explore the relationships between health status, preventive health behaviors, and healthcare utilization among non-Hispanic Black and Hispanic men aged 40 years and older (49). Therefore, using the Qualtrics panel was particularly suited to the target population, as African American and Hispanic men have historically shown lower responsiveness to health-related research participation requests (50). The questionnaire, consisting of validated scales from previous research, underwent a thorough review by external experts in the field who were not involved in the research team. Based on their feedback, the survey instrument was refined, with careful consideration given to the inclusion or exclusion of specific items, along with additional explanations for the verified scales (49, 51–55). Potential participants were required to acknowledge the information sheet by clicking “I agree” before participating in the questionnaire and participants who consented to the study completed a 105-item survey focused on health and health-related outcomes, including aspects of self-management and control (56–58).

**Figure 1:**
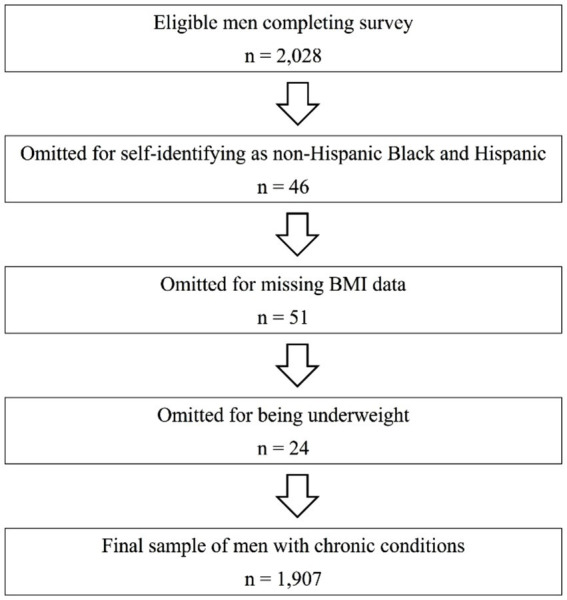
Flow diagram of participant selection.

Recruitment efforts were conducted between September and October 2019, resulting in a total of 2,028 eligible men completing the survey instrument. Of these, 46 cases were omitted due to men self-reporting they were both non-Hispanic Black and Hispanic (i.e., too small of a subgroup for meaningful comparison). Additional cases were excluded for missing body mass index (BMI) data (*n* = 51) and the small proportion of men reporting being underweight (*n* = 24). Subsequently, the final analytical sample consisted of 1,907 non-Hispanic Black and Hispanic men aged 40 years and older with one or more chronic conditions. Figure 1 displays a flow diagram for the analytic sample used in this study. This study adheres to the Declaration of Helsinki involving human subjects. Institutional Review Board approval for this study was obtained from Texas A&M University (#2018–1,684).

### 2.2 Measures

#### 2.2.1 Dependent variable

The dependent variable in this study was self-reported depressive symptoms, assessed using the Patient Health Questionnaire-2 (PHQ-2). The PHQ-2 consists of two questions regarding feelings of depression and interest in usual activities over the past 2 weeks (59, 60). Each item is rated on a 4-point Likert scale, ranging from “not at all” to “nearly every day.” Scores for the PHQ-2 range from 0 to 6 when summed. The recommended cut-point of 3 was utilized to distinguish between individuals with no depressive symptoms (scores 0–2) and those with depressive symptoms (scores 3–6) (59). No approvals or permissions are required to utilize this tool.

#### 2.2.2 Chronic disease profile and health indicators

Chronic conditions were assessed using a comprehensive self-reported checklist encompassing 15 chronic health conditions, which included: (1) asthma/emphysema/chronic breathing or lung problem; (2) arthritis/rheumatic disease; (3) cancer or cancer survivor; (4) chronic pain; (5) diabetes; (6) heart disease; (7) high cholesterol; (8) hypertension, (9) kidney disease; (10) osteoporosis; (11) obstructive sleep apnea; (12) stroke; (13) thyroid problem; (14) urinary incontinence; and (15) another chronic condition not listed. Participants were asked to indicate the conditions that applied to them from the provided list. Endorsed items were aggregated to derive a composite score reflecting the total number of chronic conditions experienced by each participant, with scores ranging from 1 to 16. Additionally, participants reported the daily number of different medications taken, with responses ranging from 0 to 6 or more. BMI was computed using participants’ self-reported height and weight, and subsequently categorized as normal weight, overweight, or obese.

Quality of life was measured using the instrument from the National Study of the Chronic Disease Self-Management Program (61, 62). It was evaluated using a rating scale ranging from 0 (indicating the worst possible quality of life) to 10 (indicating the best possible quality of life). Using items from the CDC Health-Related Quality of Life instrument, participants also reported the number of days of unhealthy physical health experienced in the past 30 day (63). This variable was dichotomized to distinguish between those experiencing frequent physical distress (14 or more days) and those experiencing no frequent distress (0 to 13 days). Similarly, participants reported the number of days during the past 30 days when poor physical or mental health prevented them from engaging in their usual activities. This variable was dichotomized to identify frequent activity limitations (14 or more days) and no frequent limitations (0 to 13 days).

#### 2.2.3 Lifestyle behaviors

Lifestyle behaviors examined were sedentary behavior (hours spent sitting per week), tobacco use, and alcohol consumption. These factors were selected because lifestyle behaviors are known modifiable risk factors that contribute to both chronic physical and mental health conditions (40, 42). Sedentary behavior was considered because prolonged sitting time has been consistently associated with adverse mental health outcomes, such as increased depressive symptoms, likely mediated by decreased physical activity levels and resultant declines in physical health and mental well-being (64, 65). The inclusion of tobacco and alcohol use was supported by literature linking substance use behaviors to increased risk for depressive symptoms and poor chronic disease management (66, 67).

In this study, participants’ weekly sitting time, smoking behavior in the past 30 days, and weekly alcohol consumption were evaluated as health behavioral factors. These factors were measured using the Behavioral Risk Factor Surveillance System (BRFSS) questionnaire (68), which is specifically designed to collect data on behavioral risk factors linked to the most common health conditions in the United States. Participants reported the approximate number of hours spent sitting per week. They also indicated whether they had used any tobacco products in the past 30 days, with responses treated as dichotomous (no or yes). Additionally, participants reported the number of alcoholic beverages consumed per week. Due to skewed responses, this variable was dichotomized to differentiate between those who consumed any alcohol weekly and those who did not.

#### 2.2.4 Perceptions about self-care barriers and healthcare frustrations

The Self-Care Barriers Scale comprises five items designed to assess participants’ agreement or disagreement using a 4-point scale (49, 69, 70). Items include the need for assistance in learning how to improve health management, financial constraints hindering health-related actions, desires to adopt healthier behaviors but feeling incapable, and challenges associated with managing multiple health conditions. Responses ranged from strongly disagree to strongly agree, with scores ranging from 5 to 20 indicating the extent of perceived barriers (52), where higher scores correspond to greater barriers. The internal consistency of this scale within the current sample was good (Cronbach’s *α* = 0.844) (69).

The Healthcare Frustrations Scale consisted of 6 items that assessed participants’ frustrations with various aspects of their medical experience, using a 3-point Likert scale ranging from “never” (scored 1) to “frequently” (scored 3) (49, 69, 70). The specific items measured frustrations related to feelings of repetitiveness in describing health conditions, confusion after healthcare appointments, desires for more doctor-patient interaction time, feelings of isolation in health management, perceptions of healthcare provider understanding, and wishes for accompaniment to medical visits by friends or family members. Participants’ responses to these 6 items were summed into a total score, with possible values ranging from 6 to 18. Higher scores indicate greater healthcare frustrations (71). The Cronbach’s alpha scale value was also good for the current sample (*α* = 0.854).

#### 2.2.5 Social engagement and support variables

Variables within this category included reliance on others for health management and social disconnectedness. The inclusion of these measures aligns with the Social Support Theory (72), which suggests that perceived availability and adequacy of support networks significantly influence mental health outcomes, including depression. The extent to which participants received necessary health-related assistance and support, as well as their reliance on such support networks, was evaluated using a 5-point scale ranging from never (0) to always (4) (52, 69, 70). To address the skewed distribution of responses, these were divided into categories of never/rarely/occasionally versus frequently/always. Reliance on ongoing assistance and support for health improvement and management was assessed across various sources, including spouses or partners, friends or relatives, individuals with similar health conditions, co-workers, healthcare professionals, faith-based organizations, community groups or clubs, and the Internet (69, 70). The internal consistency of this assessment tool, as measured by Cronbach’s alpha coefficient, was deemed good at 0.837.

The Brief Upstream Social Interaction Risk Score (U-SIRS-4) is a concise 4-item measure designed to evaluate an individual’s perception of social disconnectedness (73). Items assess feelings such as isolation from others, the ability to find companionship when desired, missing the presence of people, and participation in community groups or organizations within the past week. Response options for each item were binary, with choices of “no” and “yes,” which were subsequently coded as “no risk” (scored 0) and “risk” (scored 1) based on the directionality of each item (73). Scores were summed to generate a count variable (ranging from 0 to 4), with higher scores indicative of increased social disconnectedness (53, 63, 73).

#### 2.2.6 Sociodemographics

Sociodemographic variables encompassed several key measures, including age, race/ethnicity (categorized as non-Hispanic Black or Hispanic), educational attainment (classified as ≤high school graduate, some college/2-year degree, or ≥4-year degree), partner status (distinguished between those married or partnered and those never married, divorced, separated, or widowed), employment status (categorized as employed, not employed, retired, or disabled), the number of individuals residing in the household (inclusive of oneself), annual household income level (reported in approximate $10,000 USD increments), and residential rurality status (classified as metro or non-metro).

### 2.3 Statistical analysis

All statistical analyses were conducted using SPSS version 28. Descriptive statistics were computed for all variables of interest and compared across categories of self-reported depression. Chi-square tests were employed for categorical variables, while independent sample t-tests were utilized for continuous and count variables. Additionally, a descriptive subset analysis was conducted among the 458 men who reported depressive symptoms to characterize if anyone in their household utilized mental health services and their perceived access to mental health services.

Given the dichotomous nature of depressive symptoms, the dependent variable, logistic regression models were fitted. Three models were fitted to examine factors associated with depressive symptoms: (a) all men (*n* = 1,907); (b) non-Hispanic Black men only (*n* = 1,117); and (c) Hispanic men only (*n* = 790). Model selection for all three models was guided by stepwise regression, employing backward elimination of non-significant predictor variables. Initially, all independent variables were included in the model (i.e., sociodemographics, disease profile and health indicators, lifestyle behaviors, perceptions about self-care barriers and healthcare frustrations, and social engagement and support). Variables that did not significantly contribute to the model (using a *p*-value threshold of *p* < 0.05) were sequentially removed at each iteration (74). The final iteration retained only variables significantly associated with depressive symptoms (*p* < 0.05), providing the best fit as indicated by the likelihood ratio test. For all men, the final model was determined after 10 iterations, yielding a Nagelkerke R Square value of 0.440. For non-Hispanic Black men only, the final model was determined after 12 iterations, yielding a Nagelkerke R Square value of 0.430. For Hispanic men only, the final model was determined after 13 iterations, yielding a Nagelkerke R Square value of 0.465. Statistical significance for all analyses was set at *p* < 0.05.

## 3 Results

Table 1 contains sample characteristics compared by self-reported depressive symptom status. Of the men in this study, 24.0% self-reported depressive symptoms. On average, participants were aged 56.72 (±10.03) years. Most men (58.6%) were non-Hispanic Black, had more than a high school education (79.4%), not partnered (52.1%), and lived in urban areas (93.6%). On average, men lived in households with 2.60 (±1.64) people and had annual household incomes of approximately $60,000 (±$33,800). Over 48% of men were employed, with 29.6% being retired, 14.3% disabled, and 8% unemployed or a student. On average, men self-reported 3.53 (±2.56) chronic conditions and taking 3.39 (±2.02) medication daily. About 43% of men were obese and another 35.4% were overweight. Over 35% of participants used tobacco in the past 30 days, 60.9% consumed alcohol in a typical week, and 57.5% reported getting the help/support they needed to manage their health problems.

**Table 1:** Sample characteristics.

Variables	Total (*n* = 1,907)	No depression (*n* = 1,449)	Depression (*n* = 458)	χ^2^ or t	*p*
Patient Health Questionnaire (PHQ-2) (0 to 6)	1.55 (±1.80)	0.69 (±0.85)	4.29 (±1.13)	--	--
Race and Ethnicity				10.85	<0.001
Non-Hispanic Black	58.6%	60.7%	52.0%		
Hispanic	41.4%	39.3%	48.0%		
Age	56.72 (±10.03)	58.16 (±9.93)	52.15 (±8.93)	12.21	<0.001
Education level				14.43	<0.001
High School or Less	20.6%	19.0%	25.3%		
Some college or 2-Year degree	42.9%	42.3%	44.8%		
4-year college or more	36.5%	38.6%	29.9%		
Partner status				5.32	0.021
Married/Partnered	47.9%	46.4%	52.6%		
Unmarried/separated/divorced/widowed	52.1%	53.6%	47.4%		
Employment status				98.27	<0.001
Employed	48.1%	47.6%	49.6%		
Retired	29.6%	34.4%	14.6%		
Disabled	14.3%	11.0%	24.5%		
Unemployed/student	8.0%	7.0%	11.4%		
Number of persons in household (including self)	2.60 (±1.64)	2.47 (±1.46)	2.98 (±2.05)	−4.94	<0.001
Annual household income (~$10,000 increments)	6.02 (±3.38)	6.27 (±3.43)	5.25 (±3.10)	5.98	<0.001
Residential rurality				0.66	0.418
Urban	93.6%	93.9%	92.8%		
Rural	6.4%	6.1%	7.2%		
Number of chronic conditions (0 to 15)	3.53 (±2.56)	3.42 (±2.54)	3.87 (±2.59)	−3.30	<0.001
Asthma/emphysema/other chronic lung problem	18.8%	16.8%	24.9%	14.80	<0.001
Arthritis/rheumatic disease	29.9%	28.6%	34.1%	5.01	0.025
Cancer or cancer survivor	14.0%	15.2%	10.3%	7.00	0.008
Chronic pain	36.2%	31.3%	51.7%	63.24	<0.001
Diabetes	37.8%	38.2%	36.5%	0.46	0.496
Heart disease	13.0%	13.2%	12.2%	0.28	0.596
High cholesterol	45.6%	45.8%	44.8%	0.16	0.690
Hypertension	56.0%	56.9%	53.1%	2.13	0.145
Kidney disease	7.8%	8.6%	5.2%	5.54	0.019
Osteoporosis	6.4%	6.0%	7.9%	1.99	0.159
Obstructive sleep apnea	22.4%	20.6%	27.9%	10.71	0.001
Stroke	6.6%	6.2%	7.9%	1.53	0.216
Thyroid problem	8.8%	9.0%	8.1%	0.40	0.527
Urinary incontinence	9.6%	9.7%	9.4%	0.03	0.863
Other chronic condition	16.8%	14.5%	24.0%	22.61	<0.001
Number of medications taken daily	3.39 (±2.02)	3.33 (±2.02)	3.58 (±2.01)	−2.35	0.019
Body Mass Index				3.68	0.159
Normal Weight	21.3%	21.0%	22.5%		
Overweight	35.4%	36.6%	31.7%		
Obese	43.3%	42.4%	45.9%		
Quality of life	6.69 (±1.92)	7.21 (±1.69)	5.86 (±2.21)	12.06	<0.001
Frequent Physical Distress (past 30 days)				78.25	<0.001
0 to 13 days	85.4%	89.4%	72.7%		
14 + days	14.6%	10.6%	27.3%		
Frequent activity limitations due to health problems (past 30 days)				185.53	<0.001
0 to 13 days	88.7%	94.3%	71.2%		
14 + days	11.3%	5.7%	28.8%		
Number of hours sitting (per week)	14.89 (±11.54)	14.11 (±10.08)	17.37 (±15.00)	−4.35	<0.001
Tobacco use (past 30 days)				56.75	<0.001
No	64.9%	69.5%	50.2%		
Yes	35.1%	30.5%	49.8%		
Consume alcohol (typical week)				1.19	0.276
No	39.1%	39.8%	36.9%		
Yes	60.9%	60.2%	63.1%		
Barriers to self-care scale (5 to 20)	11.47 (±3.64)	10.67 (±3.42)	13.99 (±3.14)	−19.27	<0.001
Healthcare frustrations scale (6 to 18)	9.44 (±3.11)	8.77 (±2.77)	11.55 (±3.16)	−16.89	<0.001
Rely on others to improve and manage health/problems scale (8 to 40)	16.77 (±6.08)	16.31 (±5.69)	18.24 (±7.01)	−5.35	<0.001
Get help/support needed to manage health problems				87.41	<0.001
Never/rarely/occasionally	42.5%	36.6%	61.4%		
Frequently/always	57.5%	63.4%	38.6%		
Brief upstream social interaction risk score (U-SIRS-4) (0 to 4)	1.64 (±0.99)	1.44 (±0.86)	2.30 (±1.06)	−15.80	<0.001

When comparing participants by self-reported depressive symptoms, a significantly larger proportion of men who were Hispanic (χ^2^ = 10.85, *p* < 0.001), less educated (χ^2^ = 14.43, *p* < 0.001), not partnered (χ^2^ = 5.32, *p* = 0.021), and disabled/unemployed (χ^2^ = 98.27, *p* < 0.001) reported depressive symptoms. On average, men who reported depressive symptoms were younger (*t* = 12.21, *p* < 0.001), lived with more individuals (*t* = −4.94, *p* < 0.001), and had lower annual household incomes (*t* = 5.98, *p* < 0.001). On average, men who reported depressive symptoms had more chronic health conditions (*t* = −3.30, *p* < 0.001). Significantly larger proportions of men who self-reported asthma/emphysema/other chronic lung problems (χ^2^ = 14.80, *p* < 0.001), arthritis/rheumatic disease (χ^2^ = 5.01, *p* = 0.025), chronic pain (χ^2^ = 63.24, *p* < 0.001), obstructive sleep apnea (χ^2^ = 10.71, *p* = 0.001), and other chronic conditions (χ^2^ = 22.61, *p* < 0.001) reported depressive symptoms. Significantly smaller proportions of men who self-reported cancer or cancer survivorship (χ^2^ = 7.00, *p* = 0.008) and kidney disease (χ^2^ = 5.54, *p* = 0.019) reported depressive symptoms. On average, men who reported depressive symptoms took more medications daily (*t* = 5.98, *p* < 0.001), spent more time sitting (*t* = −4.35, *p* < 0.001), had lower quality of life (*t* = −2.35, *p* = 0.019), had more barriers to self-care (*t* = −19.27, *p* < 0.001), and had more healthcare frustrations (*t* = −16.89, *p* < 0.001). On average, men who reported depressive symptoms relied more on others to manage their health problems (*t* = −5.35, *p* < 0.001), and reported more social disconnectedness (*t* = −15.80, *p* < 0.001). A significantly larger proportion of men who had frequent physical distress (χ^2^ = 78.25, *p* < 0.001), frequent activity limitations due to health problems (χ^2^ = 185.53, *p* < 0.001), and used tobacco (χ^2^ = 56.75, *p* < 0.001) reported depressive symptoms.

In a subset analysis of the 458 men with self-reported depressive symptoms (table not reported), 54.1% reported no one in their household (including themselves) needed mental health services, 15.9% reported a household member (including themselves) needed but did not use mental health services, and 29.9% reported a household member (including themselves) needed and used mental health services. Among these 458 men, 35.2% reported their access to mental health care, if needed, was “very poor,” “poor,” or “fair.”

Table 2 contains findings from multivariable logistic regression examining factors associated with self-reported depressive symptoms among all participants. Relative to non-Hispanic Black men, Hispanic men were more likely to report depressive symptoms (OR = 1.39, *p* < 0.017). On average, for each additional year, men who were older were less likely to report depressive symptoms (OR = 0.97, *p* < 0.001). On average, for each unit increase, men who lived with more people in their household (OR = 1.13, *p* = 0.001) and took more medications daily (OR = 1.10, *p* < 0.010) were more likely to report depressive symptoms. Men who reported frequent physical distress (OR = 3.15, *p* < 0.001) and used tobacco (OR = 1.56, *p* = 0.002) were more likely to report depressive symptoms. On average, for each unit increase, men who spent more hours sitting (OR = 1.01, *p* = 0.030), had more barriers to self-care (OR = 1.16, *p* < 0.001), reported higher healthcare frustrations (OR = 1.13, *p* < 0.001), relied more on others to improve/manage health (OR = 1.04, *p* < 0.001), and had higher social disconnectedness (OR = 1.65, *p* < 0.001) had greater odds of reporting depressive symptoms.

**Table 2:** Factors associated with self-reported depression (all men) (*n* = 1,907).

Variables	*p*	OR	95% CI	
			Lower	Upper
Hispanic (vs. Non-Hispanic Black)	0.017	1.39	1.06	1.82
Age	<0.001	0.97	0.96	0.99
Number of people in household	0.001	1.13	1.05	1.22
Rural residence (vs. Urban Residence)	0.081	1.59	0.95	2.66
Number of medications Taken Daily	0.010	1.10	1.02	1.18
Quality of life (0 to 10)	<0.001	0.81	0.76	0.88
Frequent physical distress (vs. 0 to 13 days)	<0.001	3.15	2.17	4.57
Number of hours spent sitting each week	0.030	1.01	1.00	1.02
Tobacco use in past 30 days (vs. No Use)	0.002	1.56	1.18	2.05
Barriers to self-care (5 to 20)	<0.001	1.16	1.10	1.21
Healthcare frustrations (6 to 18)	<0.001	1.13	1.08	1.18
Rely on others to improve and manage health/problems (8 to 40)	<0.001	1.04	1.02	1.06
Brief upstream social interaction risk score (U-SIRS-4)	<0.001	1.65	1.43	1.89

After 10 backward stepwise iterations [Nagelkerke R Square = 0.440]. The model adjusted for sociodemographic, disease characteristics, health status, social engagement and support, and lifestyle behaviors.

Table 3 contains findings from two multivariable logistic regressions examining factors associated with self-reported depressive symptoms among non-Hispanic Black men and Hispanic men separately. Commonly across non-Hispanic Black men and Hispanic men, on average, each additional year of age reduced the odds of reporting depressive symptoms, respectively (OR = 0.96, *p* < 0.001; OR = 0.98, *p* = 0.044). Commonly across non-Hispanic Black men and Hispanic men, for each unit increase, men who reported frequent activity limitations due to health problems (OR = 2.57, *p* = 0.002; OR = 2.90, *p* < 0.001), had more barriers to self-care (OR = 1.19, *p* < 0.001; OR = 1.15, *p* < 0.001), reported higher healthcare frustrations (OR = 1.09, *p* = 0.008; OR = 1.19, *p* < 0.001), and had higher social disconnectedness (OR = 1.66, *p* < 0.001; OR = 1.65, p < 0.001) had greater odds of reporting depressive symptoms, respectively. Unique to non-Hispanic Black men, on average, for each unit increase, men who reported higher quality of life had lower odds of reporting depressive symptoms (OR = 0.76, *p* < 0.001). Non-Hispanic Black men who used tobacco were more likely to report depressive symptoms (OR = 1.58, *p* = 0.014). Unique to non-Hispanic Black men, on average, for each unit increase, men who relied more on others to improve/manage health had higher odds of reporting depressive symptoms (OR = 1.05, *p* < 0.001). Unique to Hispanic men, on average, for each unit increase, men who lived with more people in their household had greater odds of reporting depressive symptoms (OR = 1.40, *p* < 0.001), whereas Hispanic men who reported higher annual household incomes had lower odds of reporting depressive symptoms (OR = 0.94, *p* = 0.032). On average, for each unit increase, Hispanic men who spent more hours sitting had higher odds of depressive symptoms (OR = 1.03, *p* = 0.020).

**Table 3:** Factors associated with self-reported depression by race and ethnicity.

Variables	Black men only (*n* = 1,117)				Hispanic men only (*n* = 790)			
	*p*	OR	95% CI		*p*	OR	95% CI	
			Lower	Upper			Lower	Upper
Age	<0.001	0.96	0.94	0.99	0.044	0.98	0.95	1.00
Number of people in household	--	--	--	--	<0.001	1.40	1.21	1.62
Annual household income (~$10,000 increments)	--	--	--	--	0.032	0.94	0.88	0.99
Number of medications taken daily	0.055	1.10	1.00	1.21	0.084	1.10	0.99	1.22
Quality of life (0 to 10)	<0.001	0.76	0.69	0.84	--	--	--	--
Frequent physical distress (vs. 0 to 13 days)	0.089	1.59	0.93	2.70	--	--	--	--
Frequent activity limitations due to health problems (past 30 days)	0.002	2.57	1.40	4.73	<0.001	2.90	1.68	5.01
Number of hours spent sitting each week	--	--	--	--	0.020	1.03	1.00	1.05
Tobacco use in past 30 days (vs. No Use)	0.014	1.58	1.10	2.28	--	--	--	--
Barriers to self-care (5 to 20)	<0.001	1.19	1.11	1.26	<0.001	1.15	1.07	1.24
Healthcare frustrations (6 to 18)	0.008	1.09	1.02	1.16	<0.001	1.19	1.11	1.27
Rely on others to improve and manage health/problems (8 to 40)	<0.001	1.05	1.02	1.09	--	--	--	--
Brief upstream social interaction risk score (U-SIRS-4)	<0.001	1.66	1.38	2.00	<0.001	1.65	1.34	2.02
	After 12 backward stepwise iterations [Nagelkerke R Square = 0.430]				After 13 backward stepwise iterations [Nagelkerke R Square = 0.465]			

Models adjusted for sociodemographic, disease characteristics, health status, social engagement and support, and lifestyle behaviors.

## 4 Discussion

This study investigated the prevalence and determinants of self-reported depression among non-Hispanic Black and Hispanic men aged 40 years and above with chronic conditions. Nearly one-in-four men reported depressive symptoms. Hispanic men were more likely to self-report depressive symptoms compared to non-Hispanic Black men. While existing literature identifies general disparities in depressive symptoms among racial and ethnic groups, specific prevalence data and detailed mechanisms underlying these differences remain underexplored, particularly among non-Hispanic Black and Hispanic men with chronic conditions (75). While non-Hispanic Black and Hispanic men may experience some of the same barriers related to mental health, such as stigma (76, 77) and inconsistent access to healthcare (9, 78), they may also share protective factors like ethnic identity (79–81) and family support (82, 83). Although the current study did not assess variables such as acculturation (84), sub-ethnic origin (e.g., Mexican American, Puerto Rican, Cuban, and other) (75, 85, 86), or language proficiency (76), prior research suggests these factors may play a role in depression prevalence among Hispanic populations.

The subgroup analyses by race and ethnicity in this study revealed differences that could inform targeted interventions. For non-Hispanic Black men, a higher quality of life was associated with a lower likelihood of reporting depressive symptoms, suggesting that efforts to improve life satisfaction through health interventions or lifestyle changes may be particularly beneficial for this group (78). Given these findings, for non-Hispanic Black men, interventions that focus on enhancing quality of life and social engagement, such as community-based programs that foster social connections and improve self-management of chronic conditions, may be effective in reducing depressive symptoms. For Hispanic men, living with more individuals in the household was linked to an increased likelihood of depressive symptoms. This may be due to the additional stress and responsibilities that come with larger households, which can exacerbate the mental health and financial burdens (81, 87). Such findings highlight the need for interventions tailored to specific cultural and social contexts, where the family dynamic plays a central role. For instance, while increased household size might provide emotional and instrumental support in some contexts, it can also introduce stressors such as financial burdens and caregiving responsibilities, which can negatively affect mental health (81). For Hispanic men, programs that address household stressors and promote family support, while also acknowledging the role of cultural factors like familismo, could help mitigate the negative mental health impacts of larger household sizes. While these subgroup analyses help elucidate specific protective or risk factors unique to these populations and inform tailored interventions, future research utilizing larger and more diverse datasets could further explore these differences comprehensively.

Across all models in the current study, older age was associated with lower odds of reporting depressive symptoms. This trend may be due to the development of psychological resilience (88) and more adaptive coping strategies with age, which help reduce the likelihood of depression (88–91). Additionally, while not directly examined in the current study, older men with chronic conditions may have more stable and close social networks and receive support from spouses, family, or friends, which can help alleviate depressive symptoms (83, 92, 93). While these results highlight important findings, a more thorough subgroup analysis examining distinct age-related factors affecting depression among Hispanic and non-Hispanic Black men separately is necessary.

Across all study models, men who felt socially disconnected were more likely to report depressive symptoms, findings that align with existing studies (94, 95). As discussed by Santini et al. (94), perceived isolation, including feelings of loneliness, could predict both depressive symptoms and social withdrawal. Implementing formal and informal strategies to improve perceived isolation and reduce loneliness could help improve men’s mental health outcomes. For example, participating in Chronic Disease Self-Management Education workshops, which meet regularly in small groups to overcome common barriers to disease symptom management, could reduce loneliness among men with chronic conditions and help them feel more socially connected (96).

Residing with more individuals was not a protective factor in the current study, especially for Hispanic men. Men living in larger households were more likely to report depressive symptoms. The increased number of household members may lead to additional responsibilities, chores, and greater financial burden (97). These stressors can contribute to the onset or exacerbation of depressive symptoms, creating a cycle of mental health challenges within the household. Greenberg and colleagues also highlighted the potential for depression to have a spillover effect (98), where the mental health struggles of one household member can influence the well-being of others, thereby increasing overall household stress and exacerbating mental health issues for multiple individuals. These dynamic underscores the need for targeted interventions that consider the mental health of all household members, especially in high-stress environments. In this study’s subset analysis of men with depressive symptoms, despite self-reporting these symptoms, over half reported that no one in their household (themselves included) utilized mental health services, and another 15.9% went without mental health services, even when needed. Further, over one-in-three men reported they had less-than-good access to mental health care. Taken together, these findings highlight the urgency of properly diagnosing and treating depression, and reducing barriers to mental health services, among non-Hispanic Black and Hispanic men and those living within their households. Dedicated efforts are needed to identify, understand, and reduce access barriers, inclusive of inequitable systemic and structural barriers (99, 100), to improve mental health outcomes for men and their families. Such efforts are needed to improve individual access to care and enhance family-level support systems that promote mental well-being.

Barriers to self-care and reliance on others to manage their condition were also associated with more depressive symptoms, which supports the findings that showed a bidirectional relationship between depression and self-care health behaviors (101, 102). Depressive symptoms, such as loss of interest and lack of motivation, can hinder self-care efforts, while neglecting self-care can worsen depressive symptoms. For example, in our study, non-Hispanic Black and Hispanic men with depressive symptoms were found to be more sedentary compared to those without such symptoms. Sedentary behaviors may have increased the risk of depression, or depressive symptoms may have led to increased sedentary behaviors (7, 64–67, 103–105). Cultural values of self-reliance and machismo among non-Hispanic Black and Hispanic men can also explain the association between reliance on others and depression (106, 107). Research has shown that masculine ideals, such as strength, autonomy, and control, often discourage expressions of vulnerability or dependence on others (108). This cultural framework may influence the way men perceive self-care behaviors, such as taking medications or seeking support. Swetlitz et al. (76) proposed redefining depression care to affirm masculine identity, where family and healthcare providers could help reframe self-care behaviors (e.g., taking medications) or reliance on others to assert masculine values (e.g., strength, autonomy, control). Specifically, emphasizing strength, autonomy, and control in self-care practices may reduce stigma and encourage engagement with treatment (76). Furthermore, healthcare-related frustrations, which are common among individuals facing barriers to self-care (72), were also associated with depressive symptoms. Effective patient-provider communication should emphasize maintaining a culturally sensitive dialogue that promotes shared decision-making to alleviate these frustrations, while affirming the male patient’s sense of masculinity (76). Public health professionals, including community health workers, can also play a vital role by providing mental health literacy education to non-Hispanic Black and Hispanic men to empower them to access mental health care for the screening and treatment of depression (109). Mental health literacy campaigns, similar to England’s Every Mind Matters (110, 111), could be organized for non-Hispanic Black and Hispanic men with chronic conditions to raise awareness of mental health and promote self-management of depressive symptoms. To enhance mental health care for non-Hispanic Black and Hispanic men with chronic conditions, policymakers should advocate for culturally responsive leadership in mental health organizations (112). This strategy would promote the recruitment and retention of a diverse mental health workforce, particularly from non-Hispanic Black and Hispanic backgrounds, who can more effectively address the unique needs of their communities.

In bivariate analyses, men with depressive symptoms averaged more comorbid chronic conditions, and larger proportions of men who self-reported certain chronic conditions (i.e., asthma/emphysema/other chronic lung problems, arthritis/rheumatic disease, chronic pain, obstructive sleep apnea) reported depressive symptoms. While the number of self-reported chronic conditions was not significantly associated with depressive symptoms in multivariate analyses, it is recognized that disease comorbidity may shape the onset, recognition, and treatment of depressive symptoms. Symptomatic chronic conditions (e.g., chronic pain, obstructive sleep apnea) can increase the risk for depression (22, 113, 114). These physical symptoms can mask or mirror depressive symptom (115, 116), thus complicating depression diagnosis and treatment (117–119). Those with comorbid chronic conditions and depression may diminish treatment adherence and self-care behaviors (120–123). As such, the interplay between physical and mental health may require a multi-disciplinary and biopsychosocial approach to optimize health outcomes and prevent potentially severe disease complications (124–126).

The current study has limitations warranting acknowledgement. First, it employed a cross-sectional design, which restricts the ability to infer causality over time. Second, only self-reported data were collected, potentially affecting the validity and reliability of the findings. Third, the internet-based survey may have created some selection bias, where those with less internet proficiency or limited internet access may be underrepresented. With the opt-in nature of the Qualtrics panel, it is difficult to determine if those who chose to participate differ systematically from those who were invited but declined to participate in the study. Fourth, the brief PHQ-2 was utilized for assessing depressive symptoms, but it may have underestimated or oversimplified the prevalence and severity of depression symptomatology compared to using longer versions of the tool (e.g., PHQ-8 or PHQ-9). While the PHQ-2 is a well-known screener for depression symptoms, additional clinical efforts are needed to properly diagnose depression among non-Hispanic Black and Hispanic men, including the use of the full PHQ-9. Fifth, because our sample consisted exclusively of non-Hispanic Black and Hispanic men aged ≥40 years with chronic conditions, the findings are not generalizable to women, younger individuals, or those without chronic illness. Although our analysis provided important insights into shared determinants of depressive symptoms among minority men with chronic conditions, future research should assess whether these associations hold across more diverse populations. Sixth, while participants represented all four census quadrants in the United States, geographic heterogeneity across regions was not explored. The United States is diverse, and experiences related to race, ethnicity, social engagement, and lifestyle behaviors may substantially differ based on geographic region (e.g., West Coast, East Coast, Deep South). Future research should incorporate geographic variables to better contextualize findings, which would allow for a more nuanced understanding of depressive symptomatology among non-Hispanic Black and Hispanic men with chronic conditions. Seventh, some of our variables were dichotomized, including depressive symptoms measured with the PHQ-2, which may overlook certain nuances across variable categories and oversimplify the complexity of depressive symptomology. Although variables were dichotomized based on frequency distributions and/or other well-documented thresholds, binary categorizations for depression may mask subtler distinctions in symptom severity and impact, and future studies could benefit from considering a more nuanced approach to assessing depression, potentially using continuous measures or multi-level categorization. Finally, the timing of data collection, which occurred in late 2019, prior to the COVID-19 pandemic, may be a shortcoming to the current study. Given the documented impacts of the pandemic on mental health, including disproportionate effects on racial and ethnic minority groups, the current findings may not reflect subsequent shifts in depressive symptoms, social engagement, or lifestyle behaviors that occurred due to pandemic-related social and economic disruptions. Thus, caution should be exercised when generalizing these findings to post-pandemic contexts.

In conclusion, this study highlights significant racial/ethnic disparities in the prevalence of self-reported depressive symptoms among non-Hispanic Black and Hispanic men aged 40 years and above with chronic conditions. Several factors were identified as determinants of depressive symptoms, including social disconnectedness and healthcare frustrations, underscoring the importance of tailored interventions to empower non-Hispanic Black and Hispanic men with chronic conditions to seek help and engage in self-care to manage their depression.

## Data Availability

The raw data supporting the conclusions of this article will be made available by the authors, without undue reservation.
